# Genomic analysis of dibenzofuran-degrading *Pseudomonas veronii* strain Pvy reveals its biodegradative versatility

**DOI:** 10.1093/g3journal/jkaa030

**Published:** 2020-12-09

**Authors:** Eglantina Lopez-Echartea, Jachym Suman, Tereza Smrhova, Jakub Ridl, Petr Pajer, Michal Strejcek, Ondrej Uhlik

**Affiliations:** 1 Department of Biochemistry and Microbiology, Faculty of Food and Biochemical Technology, University of Chemistry and Technology, Prague, Technicka 3, 166 28 Prague 6, Czech Republic; 2 Department of Genomics and Bioinformatics, Institute of Molecular Genetics, Academy of Sciences of the Czech Republic, Videnska 1083, 142 40 Prague, Czech Republic; 3 Division of Animal Evolutionary Biology, Department of Zoology, Faculty of Science, Charles University in Prague, Vinicna 7, 128 44 Prague, Czech Republic; 4 Military Health Institute, Ministry of Defence of the Czech Republic, U Vojenske nemocnice 1200, 169 02 Prague 6, Czech Republic

**Keywords:** *Pseudomonas veronii* strain Pvy, biodegradation, dioxygenase, dibenzofuran, whole-genome sequencing, nanopore technology, denitrification, organic phosphate mineralization, heavy-metal tolerance

## Abstract

Certain industrial chemicals accumulate in the environment due to their recalcitrant properties. Bioremediation uses the capability of some environmental bacteria to break down these chemicals and attenuate the pollution. One such bacterial strain, designated Pvy, was isolated from sediment samples from a lagoon in Romania located near an oil refinery due to its capacity to degrade dibenzofuran (DF). The genome sequence of the Pvy strain was obtained using an Oxford Nanopore MiniION platform. According to the consensus 16S rRNA gene sequence that was compiled from six 16S rRNA gene copies contained in the genome and orthologous average nucleotide identity (OrthoANI) calculation, the Pvy strain was identified as *Pseudomonas veronii*, which confirmed the identification obtained with the aid of MALDI-TOF mass spectrometry and MALDI BioTyper. The genome was analyzed with respect to enzymes responsible for the overall biodegradative versatility of the strain. The Pvy strain was able to derive carbon from naphthalene (NP) and several aromatic compounds of natural origin, including salicylic, protocatechuic, *p*-hydroxybenzoic, *trans*-cinnamic, vanillic, and indoleacetic acids or vanillin, and was shown to degrade but not utilize DF. In total seven loci were found in the Pvy genome, which enables the strain to participate in the degradation of these aromatic compounds. Our experimental data also indicate that the transcription of the NP-dioxygenase α-subunit gene (*ndoB*), carried by the plasmid of the Pvy strain, is inducible by DF. These features make the Pvy strain a potential candidate for various bioremediation applications.

## Introduction

The massive production and frequent use of persistent organic pollutants (POPs) in the last century represents a historic environmental burden. Most POPs are halogenated and frequently chlorinated, and their degradation rates are associated with the degree of chlorination. Not only are POPs persistent, but also they are toxic, bio-accumulative, can volatilize and therefore be transported over long distances from the source of emission (Lavric *et al.* 2004). In humans and other vertebrates, several POPs have proven to be a risk factor for cancer, immune deficiency, nervous system disorders, endocrine disruption, decreased respiratory functions, skin rashes, liver damage, and adverse cardiovascular effects [Wittich *et al.* 1992; Agency for Toxic Substances and Disease Registry (ATSDR) 1998; Srogi 2008; World Health Organization (WHO) 2014].

The most common approach for the remediation of polluted environments is physico-chemical treatments, with vapor stripping, thermal desorption, soil washing, incineration, pyrolysis, and landfilling being primarily used (Cunningham *et al.* 1995; Dua *et al.* 2002). However, these approaches are often economically demanding and difficult to execute. Bioremediation and phytoremediation, which rely on the ability of microorganisms and plants to degrade pollutants, on the other hand, are often a cost-effective and environmental friendly alternative to physico-chemical treatments (Liu and Suflita 1993; Schnoor *et al.* 1995; Macek *et al.* 2000; Macková *et al.* 2006; Macek *et al.* 2009). One of the major advantages of bioremediation is the possibility of the complete mineralization of contaminants (Tomei *et al.* 2013; Leewis *et al.* 2016). Although numerous microbial populations have the metabolic capacity to degrade a broad range of xenobiotics, the biodegradation mechanisms of many of these compounds are not fully understood. Soils and sediments in particular harbor abundant and functionally diverse populations due to numerous microsites and gradients, which can sustain bioremediation (Leigh *et al.* 2015; Stiborová *et al.* 2017). Among other taxa, *Pseudomonas* species have been often associated with the degradation of several anthropogenic compounds, including pesticides, pentachlorophenol, polyaromatic hydrocarbons, polychlorinated biphenyls (PCBs), polychlorinated dibenzo-*p*-dioxins and dibenzofurans (PCDD/Fs), and others (Hong *et al.* 2004; Koubek *et al.* 2013; Wasi *et al.* 2013; Wald *et al.* 2015; Ridl *et al.* 2018).

Rieske non-heme iron dioxygenases are generally responsible for catalyzing the first reaction in the aerobic degradation pathway of aromatic POPs, which is followed by cleaving dioxygenases involved in the opening of the aromatic ring (Hiraishi 2003; Field and Sierra-Alvarez 2008; Lopez-Echartea *et al.* 2016). The aim of this study was to analyze the genome of *Pseudomonas veronii* strain Pvy, which was isolated through its ability to degrade dibenzofuran (DF), with respect to aromatic ring-hydroxylating dioxygenases (ARHD) and other enzymes responsible for the biodegradative versatility of the strain. Furthermore, the induction of Pvy-borne naphthalene (NP) dioxygenase by DF was tested in order to gain deeper insight into the potential of the strain to degrade DF, and potentially PCDD/Fs.

## Materials and methods

### Strain description and cultivation conditions

The Pvy strain was isolated from sediment samples from a lake in Romania located near an oil refinery (Wald *et al.* 2015) for its capacity to degrade DF. Briefly, a 10% suspension of the sediment was prepared in 50 mL 1% (wt/vol) sodium pyrophosphate solution, incubated for 2 h at 28°C on a rotary shaker at 130 rpm, followed by a series of 10-fold dilutions to extinction in 0.85% (wt/vol) sodium chloride solution and the plating of 100 µl onto a solid mineral medium (Uhlík *et al.* 2011). A few crystals of DF (97% purity; Sigma-Aldrich, USA) were provided in the lid to allow for evaporation and the growth of bacterial cultures on the vapors of DF during incubation at 28°C for 72 h. Selected bacterial colonies producing a yellow zone of coloring were reinoculated onto a Plate Count Agar (PCA, Oxoid, UK) for further identification with the aid of MALDI-TOF mass spectrometry and MALDI BioTyper as described previously (Strejček *et al.* 2018). The DF-degrading bacterial culture, designated Pvy, was preserved over the long term in 25% glycerol stocks at −80°C.

### Genome sequencing

For DNA isolation, the culture was grown overnight on PCA at 28°C. Genomic DNA was isolated with a FastDNA SPIN Kit (MPBio, USA) following the manufacturer’s protocol. DNA libraries were prepared with a rapid sequencing kit (Oxford Nanopore) and sequenced on a Nanopore MinION instrument (FLO-MIN106 flow cell). In total, 193 782 Nanopore reads (average read length, 7961 bp; total number of bases, 1 542 749 538) were generated, corresponding approximately to a 200× sequencing depth. Canu software v1.7 (Koren *et al.* 2017) was used to assemble the reads into continuous genome sequences. The draft assembly was error-corrected by running Racon v1.3.3 twice (Vaser *et al.* 2017) using minimap2 (Li 2018) to align the input nanopore reads to the draft assembly. Two rounds of nanopolish v0.10.2 (Loman *et al.* 2015) were performed to further correct the assembly with the fix-homopolymer option turned on and min-candidate-frequency option set to 0.1. The BWA package (Li 2013) was used to map the input nanopore reads against the Racon-corrected assembly. Unless stated otherwise, all the above-mentioned software was run with default parameters, following the authors’ instructions, and using command prompt under a linux operation system. As the resulting assembly contained a large number of frameshifts, a sequence correction was applied as described by Arumugam *et al.* (2019). Briefly, the assembly (nucleotides) was searched against the bacterial RefSeq protein database (O'Leary *et al.* 2015) using the DIAMOND v0.9.30 blastx command in *frameshift mode* with the *range-culling* argument (Buchfink *et al.* 2015); the frameshift corrected reads were recovered from the DIAMOND output file using MEGAN Community Edition suite v6.18.5 (Huson *et al.* 2016). Both the assembly and the frameshift correction workflows are available through complementary scripts deposited in the project GitHub repository (github.com/strejcem/Pseudomonas_veronii_Pvy).

A further manual round of frameshift correction was carried out on proteins that were found to be <0.9 or >1.1 times the length of their closest match to RefSeq protein DB, during which obvious frameshifts located in homopolymer regions were corrected, while substitutions leading to premature STOP codons were left unchanged, as substitution errors are much less likely to occur than indels (Tyler *et al.* 2018) and naturally occurring pseudogenes were considered a possibility. Both automatic and manual frameshift curation was handled by only inserting one or two ambiguous nucleotides and never deleting a nucleotide, making the corrections trackable. Finally, NCBI Prokaryotic Genome Annotation Pipeline (Tatusova *et al.* 2016) was used to annotate the genome. The genome data were deposited into NCBI repositories as BioProject record PRJNA529862.

For taxonomic purposes, the OrthoANI was calculated between the genomic DNA of the Pvy strain and related strains using the OrthoANI algorithm (Lee *et al.* 2016) in EzBioCloud (Yoon *et al.* 2017).

The putative role of genes in the genome of the Pvy strain was assessed through a BLAST search of predicted protein sequences against UniProtKB/Swiss-Prot and Reference Protein (RefSeq) databases, comparison with the literature, and the KEGG database of reference pathways of aromatics degradation. The protein sequences of ARHDs, whose substrate specificities were reported in valid publications, were retrieved from the GenBank database and aligned by Muscle, together with predicted protein sequences of Pvy-borne ARHDs. The phylogenetic tree was constructed using the Maximum Likelihood method and the JTT matrix-based model (Jones *et al.* 1992) in MEGA X (Kumar *et al.* 2018).

### Utilization/degradation of aromatic substrates

The ability of the Pvy strain to utilize aromatic compounds, since putative genes to degrade them were identified in the genome, was tested as follows: a 0.22-µm filter-sterilized stock solution of DF, NP, biphenyl (BP), carbazole, salicylic acid, protocatechuic acid, indolacetic acid, vanillic acid, vanillin, *trans*-cinnamic acid, caffeic acid (all purchased from Sigma-Aldrich), and ferulic acid (purchased from Fluka), all at >97% purity and dissolved in molecular biology-grade ethanol were pipetted into 100 ml pre-sterilized flasks and the ethanol was allowed to evaporate. Pvy was inoculated into LB medium and cultivated overnight at 28°C/130 rpm, harvested by centrifugation (10 min at 8000 × *g*), washed twice and resuspended in an equal volume of mineral salt medium [MSM, composition per liter of deionized water: Na_2_HPO_4_·12H_2_O 11.0 g, KH_2_PO_4_ 2.7 g, (NH_4_)_2_SO_4_ 1 g, MgSO_4_·7H_2_O 0.2 g, FeSO_4_·7H_2_O 0.02 g, and Ca(NO_3_)_2_ 0.03 g, pH 7] (Fraraccio *et al.* 2017) to an initial optical density at 600 nm (OD_600_) of 0.05. This starting culture was then distributed in aliquots of 15 ml into flasks with individual SPMs, and the cultures were cultivated at 28°C/130 rpm for up to 6 days.

To analyze the depletion of DF by Pvy, a similar modified resting cell assay procedure was employed to that in Garrido-Sanz *et al.* (2020). Briefly, an overnight grown bacterial culture in LB medium was harvested by centrifugation, washed twice with MSM and diluted with MSM to a final OD_600_ of 1. The resultant cell suspension was then distributed in 3 ml aliquots into 15 ml glass microcosms, supplemented with 15 µl of DF solution (0.1 M in dimethylsulfoxide, the final concentration of DF in microcosms was 0.5 mM) and sealed. In addition to the Pvy cell suspension and abiotic control (MSM without cells), microcosms containing autoclaved (121°C, 15 min) Pvy cell suspension were prepared as a control of the biomass interference; all samples were prepared in quadruplicates. Microcosms were then incubated at 28°C/130 rpm for 24 h. Upon incubation, 3 ml of methanol was added to the microcosms, cell suspensions were sonicated for 5 min and cells were removed by microfiltration (0.2 µm). The DF content in the samples was then determined with an HPLC NexeraXR system with an SPD-M20A UV/VIS diode array detector (Shimadzu), using the following protocol: 0–2 min—20% methanol, 2–3.5 min—gradient of 20–95% methanol, 3.5–5.5 min—95% methanol, 5.5–7 min—gradient of 95–20% methanol, 7–8 min—20% methanol; mobile phase flow 0.8 ml/min. The separation proceeded in a Luna^®^ Omega Polar C18 column (Phenomenex) at 25°C. DF was monitored at 220 nm; under the conditions applied, DF was eluted at approximately 6.67–6.69 min. To evaluate DF residual concentration in different treatments, average areas under peak values were tested for differences by ANOVA with *post hoc* Tukey’s test using R project (R Core Team 2017).

### Induction of ARHD dioxygenase gene transcription by DF

Induction of the transcription of ARHD large subunit-encoding genes by DF in the Pvy strain was assessed by employing quantitative PCR (qPCR) using cDNA prepared from total RNA from Pvy cells exposed to DF. First, the growth curve of the Pvy strain was determined in MSM amended with 0.5% (wt/vol) sodium acetate (Sigma-Aldrich, USA) as follows: 50 ml of microbial cultures with a starting optical density at 600 nm (OD_600 nm_) of 0.025 were cultivated in a 250-ml Erlenmeyer flask on a rotary shaker (120 rpm) at 28°C in three independent biological replicates, and the growth was monitored on an hourly basis by measuring OD_600_. Second, Pvy liquid cultures were cultivated for 17 h corresponding to the mid-log phase as determined from growth curves, harvested by centrifugation (10 min at 8000 × *g*), washed twice with MSM and resuspended in MSM to OD_600_ = 1. Stock 0.03 M solution of DF in ethanol was pipetted into an empty 100-ml Erlenmeyer flask and the ethanol was allowed to evaporate, leaving behind the required amount of DF. Subsequently, 10-ml aliquots of prepared cell suspensions were added into the flasks and incubated at 28°C on a rotary shaker (120 rpm). Upon exposure to DF, bacterial cells from 3 ml of the culture were harvested by centrifugation (5 min at 5,000 × *g*) and stored for subsequent RNA isolation at −80°C. The DF-exposed Pvy cell samples were prepared in quadruplicates, starting from the cultivation of the strain.

RNA isolation was performed using the RNeasy Kit (Qiagen, USA) according to the standard protocol. Concentration of RNA and RNA integrity number (RIN) were determined using Bioanalyzer RNA 6000 Nano assay (Agilent, USA). Only samples with RIN values >7 were subjected to further analyses. Isolated RNA (2000 ng) was treated with 2 U of DNase (Thermo Fisher Scientific, USA) in 50 μl of total reaction volume. After the DNAse treatment, RNA concentration and RIN were measured again, and the absence of leftover contaminating DNA was checked using 16S rRNA gene-targeted qPCR (see further). Finally, a reverse transcription (RT) reaction was performed employing M-MuLV Reverse Transcriptase (200,000 U/ml; New England Biolabs, USA) according to the manufacturer’s instructions starting with 150 ng of RNA per 20 μl of total reaction volume.

All qPCRs were performed in a CFX96 Real-Time System (Bio-Rad, USA) using KAPA SYBR FAST qPCR Master Mix (2×) (Kapa Biosystems, USA). The PCR was performed in a final volume of 15 µl with KAPA SYBR FAST qPCR Master Mix (2×) containing 0.02 U/µl of polymerase, 0.3 µM of each primer (Sigma-Aldrich) and template genomic DNA or cDNA. The 16S rRNA gene (or its cDNA) was amplified using the universal primers 519F 5′-GTGYCAGCMGCNGCGG-3′ (Fraraccio *et al.* 2017) and 803R 5′-CTACCRGGGTATCTAATCC-3′ (Pei *et al.* 2010). The following sets of in house-designed primers were used for the quantitation of the transcripts of ARHD large subunits genes found in the Pvy genome, specifically *ndoB—*465F (5′-CTTCAAGGTATGGCACCCGA-3′) and 737R (5′-ACGACGCCAGGATAAACCTC-3′), ard1—427F (5′-AGCCAGTTCGACAAGTCCAG-3′) and 629R 5′-CACTCAATGGTGGAGCGGAT-3′, and ard2—397F (5′-CTCACCGACGATCAGCTCAA-3′) and 584R (5′-TCGGCGAAGATCCAGTTGAC-3′). The final concentration of each primer was 1.6 µM. The cycling program started with a 5-min denaturation of DNA at 95°C, followed by 30 cycles of 10 s at 95°C, 10 s at the corresponding annealing temperature, 8 s at 72°C, and a final extension of 5 min at 72°C. The annealing temperatures used were 58°C for the 16S rRNA gene, 61°C for *ndoB*, 66.5°C for *ard1*, and 63.5°C for *ard2*. Agarose gel electrophoresis, melting curve analysis, and Sanger sequencing of amplified qPCR products were used to check the specificity of the qPCR. All qPCR measurements were performed in technical triplicates and with a calibration curve constructed using serial dilutions of genomic DNA of the strain. The statistical significance of the induction experiments was tested using R project (R Core Team 2017) and pairwise *t*-tests, which were performed to compare the control (no inducer present) with DF-induced samples. Multiple *P*-values were adjusted using the Benjamini and Hochberg method (Benjamini and Hochberg 1995).

### Data availability

The strain is available upon request. The supplementary material file contains Supplementary Table S1, which shows the loci in the genome of the Pvy strain that are putatively involved in the metabolism of aromatic compounds including aromatic pollutants, Supplementary Table S2, which summarizes the utilization of aromatic substrates by Pvy strain, and supplementary text on the implications from genome analysis for heavy-metal tolerance and nutrient metabolism. The genome data were deposited into NCBI repositories as BioProject record PRJNA529862. The assembly and frameshift correction workflows are available through complementary scripts deposited in the project GitHub repository at github.com/strejcem/Pseudomonas_veronii_Pvy.

Supplementary material is available at https://gsajournals.figshare.com DOI: https://doi.org/10.25387/g3.13311809.

## Results and discussion

### Genomic analysis of the Pvy strain

The genome of the Pvy strain is composed of one chromosome and one plasmid, the total size of which is 7 305 203 bp with a total of 6704 genes, of which 6220 are coding genes. There are 94 RNA genes, of which 7 encode for 5S rRNAs, 6 for 16S rRNAs, 6 for 23S rRNAs, 71 for tRNAs, and 4 for ncRNAs. Detailed information on the genome of Pvy is provided in Tables 1 and 2.

**Table 1:** Genome project information

MIGS ID	Property	Term
MIGS 31	Finishing quality	Finished
MIGS-28	Libraries used	Rapid sequencing library (SQL-RBK004)
MIGS 29	Sequencing platforms	Oxford Nanopore MiniION
MIGS 31.2	Fold coverage	200
MIGS 30	Assemblers	Canu v. 1.7.1
MIGS 32	Gene calling method	GeneMarkS-2+
	Locus Tag	E4167
	Genbank ID	CP039631-CP039632
	GenBank Date of Release	29-JAN-2020
	GOLD ID	Gs0143776
	BIOPROJECT	PRJNA529862
	Project relevance	Bioremediation, degradation of aromatic compounds

**Table 2:** Genome statistics

Attribute	Value
Genome size (bp)	7 305 203
GC content (%)	60.67
DNA scaffolds	2
Total genes	6704
Protein coding genes	6220
RNA genes	94
Pseudo genes	390

Given that the nanopore technology, which was used for this genome sequencing, is known to be more prone to less randomly distributed errors than other sequencing technologies currently available on the market (Goldstein *et al.* 2019), two rounds were run of Racon v1.3.3 (Vaser *et al.* 2017) and nanopolish v0.10.2 (Loman *et al.* 2015) with the fix-homopolymer option turned on. After such processing, there were a total of 2562 potentially interrupted ORFs. Although some of these ORFs might have been real pseudogenes, in high-quality assembled genomes their numbers typically do not exceed 200 (Stewart *et al.* 2019), so further manual curation was performed as described in the Materials and methods section. The final number of detected potentially interrupted ORFs was 433; thus, an 83% reduction was achieved. The resulting genome consists of a single 7 110 596-bp-long chromosome and one 194 607-bp-long circular plasmid.

The consensus 16S rRNA gene sequence compiled from six 16S rRNA gene copies contained in the Pvy genome had a 99.79% similarity to the 16S rRNA gene of *P. veronii* CFML 92-134^T^, followed by *Pseudomonas extremaustralis* 14-3^T^ with 99.73% similarity and *Pseudomonas grimontii* CFML 97-514^T^ with 99.59% similarity, while OrthoANI between the genomic DNA of the Pvy strain and the type strain of *P. veronii* was 98.16%. OrthoANI with the other closest *Pseudomonas* species was below 90% (specifically 89.19% with *P. extremaustralis* 14-3^T^ and 88.22% with *P. grimontii* CFML 97-514^T^), thus corroborating the affiliation of the Pvy strain with the species *P. veronii*, which was also confirmed with the aid of MALDI-TOF mass spectrometry and MALDI BioTyper performed according to Strejček *et al.* (2018).

The overall characteristics of *P. veronii* were published by Ajithkumar *et al.* (2003) and Elomari *et al.* (1996). It is a ubiquitous bacterial species whose members have been isolated from a wide variety of different environments, including natural mineral waters (Elomari *et al.* 1996), contaminated soil (Nam *et al.* 2003; Junca and Pieper 2003), contaminated sludge (Ajithkumar *et al.* 2003), contaminated sediment (Wald *et al.* 2015), and biotrickling filter cleaners (Onaca *et al.* 2007). Finally, *P. veronii* stains have been isolated from the roots of grapevine plants (Montes *et al.* 2016), implicating their role as endophytes. As for its common occurrence in contaminated environments, *P. veronii* has been previously described as a potent degrader of many anthropogenic pollutants, including BTEX (Junca and Pieper 2003; Morales *et al.* 2016), pentachlorophenol (Nam *et al.* 2003), NP (Wald *et al.* 2015), and alkylphenols up to a chain length of six carbons (Ajithkumar *et al.* 2003). In addition, *P. veronii* strains are often heavy-metal tolerant and are capable of denitrification and the mineralization of organic phosphate (de Lima-Morales *et al.* 2013; Montes *et al.* 2016). With respect to the origin of the Pvy strain as well as its isolation as a DF-degrading bacterium, here we focus on its biodegradative potential, while the implications from the genome analysis in terms of heavy-metal tolerance and nutrient metabolism are provided in the Supplementary material.

### Utilization of aromatic substrates by the Pvy strain

To demonstrate its metabolic versatility, the utilization of a range of aromatic substances by the Pvy strain was assessed, the selection of which was based on the composition of the original sludge from which it was isolated, as well as on the genomic context of Pvy (see below). As shown in Table 3, the Pvy strain was able to utilize NP, salicylic acid, protocatechuic acid, *p*-hydroxybenzoic acid, *trans*-cinnamic acid, vanillic acid, vanillin, and indoleacetic acid, but not DF, carbazole, BP, ferulic acid, or caffeic acid.

**Table 3:** Utilization of aromatic substrates by Pvy strain

Growth substrate	Growth substrate concentration		
	5 mM	10 mM	15 mM
Dibenzofuran^a^	−	−	−
Naphthalene	+	++	+++
Carbazole	−	−	−
Biphenyl	−	−	−
Salicylic acid	++	++	−
Protocatechuic acid	++	+++	+++
*p*-Hydroxybenzoic acid	++	++	+++
Vanillic acid	++	++	++
Vanillin	+	++	++
Ferulic acid	−	−	−
*trans*-Cinnamic acid	−	−	+++
Caffeic acid	−	−	−
Indoleacetic acid	++	++	+++

The maximum growth recorded throughout the cultivation period of 6 days is shown. Legend: (−) no observable growth; (+) maximum OD_600 nm_ < 0.3; (++) 0.3 < OD_600 nm_ < 0.8; (+++) OD_600 nm_ > 0.8; results reported are from two independent experiments. Exact values are reported in Supplementary Table S2.

a Dibenzofuran was degraded by Pvy strain as assessed by the resting cell assay.

### DF degradation

The Pvy strain was isolated based on its ability to produce yellow coloration zones when grown on plates in the presence of DF; nevertheless, the strain was not able to utilize this substrate as a sole carbon source in liquid cultures (Table 3). Therefore, the ability of the strain to degrade DF was assayed by resting cell assay. Upon co-incubation with DF, the Pvy-bearing microcosms produced a yellow coloration similarly to that observed in the agar plate cultures. As analyzed by HPLC, the final DF concentration in the microcosms with Pvy cells was (0.20 ± 0.07) mM, which corresponded to 42% (significantly lower, *P*-value <0.001) of the amount in the microcosms with autoclaved Pvy cells [(0.49 ± 0.02) mM]; no significant depletion (*P*-value >0.1) was observed for autoclaved Pvy cells compared to abiotic controls, showing the matrix effect of Pvy biomass during DF analysis to be negligible.

### Implications from genome analysis: degradation of aromatic compounds

**Figure 1:**
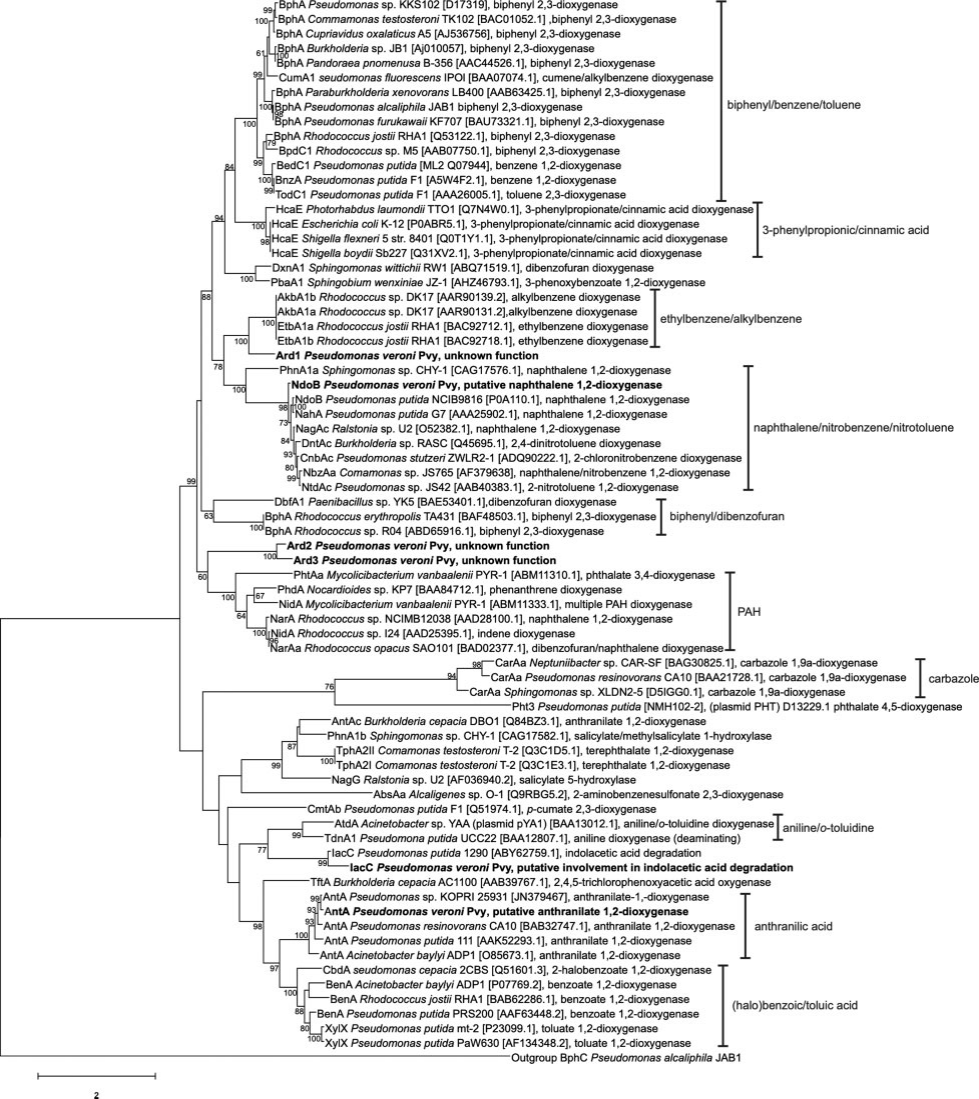
Phylogenetic tree showing relationships among ARHDs with assigned substrate specificity (the list might not be complete). The ARHDs found in Pvy strain are shown in bold, and substrates shared in clades are indicated. The analysis involved 73 amino acid sequences, whose Genbank accession numbers are indicated in brackets. Bootstrapping was used to test the tree topology (500 bootstraps, only values >70% are shown). A discrete Gamma distribution was used to model evolutionary rate differences among sites (five categories). The tree is drawn to scale, with branch lengths corresponding to the number of substitutions per site. There were a total of 551 positions in the final dataset.

The annotated genome of the Pvy strain was analyzed with respect to the presence of genes potentially involved in the degradation of aromatic compounds, including xenobiotics, with a particular emphasis on genes encoding putative ARHDs. To help elucidate the functions of putative ARHDs found in the Pvy genome, a tree showing their phylogeny was constructed along with ARHDs with assigned substrate specificity (Figure 1).

In total, seven loci were found on the chromosome of the Pvy strain for which putative roles in the degradation of aromatic compounds could be assigned based on *in silico* analyses, as shown in Supplementary Table S1. Coding sequences found within the first locus, here designated as *pca I*, exhibited high similarity with the coding sequences of enzymes from the protocatechuate branch of the β-ketoadipate pathway, including intradiol-cleaving two-component protocatechuate 3,4-dioxygenase encoded by the genes *pcaHG*, and enzymes enabling the further utilization of a protocatechuate cleavage product, 3-carboxy-*cis*,*cis*-muconate (Zylstra *et al.* 1989; Harwood and Parales 1996; Shen and Liu 2005). Environmental bacteria use the protocatechuate branch of the β-ketoadipate pathway for the degradation of various aromatic pollutants as well as natural aromatic compounds. Apart from the *pca I* locus, another copy of *pcaHG* genes was present elsewhere in the Pvy genome accompanied by the putative regulatory protein gene *pcaQ*. The ability of the Pvy strain to grow using protocatechuic acid as a sole carbon source was also proven in this study (Table 3).

Within the second region here designated as *hmg*, three putative enzymes were encoded which mediate the degradation of homogentisate to fumarate and acetoacetate. Homogentisate is an intermediate of the bacterial metabolism of ethylbenzene, styrene, and other pollutants, and at the same time, it represents both the precursor for tyrosine and phenylalanine biosynthesis and a product of their catabolism (Arias-Barrau *et al.* 2004). For that reason, the potential involvement of *hmg*-locus-borne genes in the degradation of aromatic xenobiotics is not certain.

ORFs from the *kyn/ant/cat* region encode putative enzymes enabling the transformation of tryptophan to anthranilate, including tryptophan 2,3-dioxygenase, kynurenine formamidase, and kinureninase (Kurnasov *et al.* 2003). The further catabolism of anthranilate is likely enabled by anthranilate 1,2-dioxygenase and the catechol branch of the β-ketoadipate pathway (*ortho*-cleavage) encoded by genes carried within this locus yielding 3-oxoadipate enol-lactone that can be further utilized by *in trans*-encoded enzymes, *e.g.* from the *pca* region.

The *dmp/cat* region bears putative genes encoding enzymes, which funnel phenol and possibly related phenolic compounds to intermediate metabolism through the catechol *meta*-cleavage pathway. In addition to this pathway, a short-chain alcohol dehydrogenase family protein is encoded within this locus, which provided no significant hits in databases; therefore, its role in phenol/phenolics degradation remains unknown.

The *dmp/ard1* region encodes a putative Rieske-type ARHD of unknown specificity, whose predicted α-subunit exhibits only low sequence similarity with homologous proteins included in the UniProt/Swiss-Prot database (the highest score of 56% similarity was with the BP 2,3-dioxynase subunit α from *Rhodococcus jostii* RHA1). Moreover, adjacent genes encoding for the unknown putative enzymes aromatic compound-*cis*-diol dehydrogenase and aromatic ring-opening dioxygenase are present in the *dmp/ard1* region. The remaining genes encode the catabolism of 2-hydroxymuconate semialdehyde, an intermediate of catechol *meta*-cleavage. According to its position in the phylogenetic tree, the *ard1*-corresponding protein sequence is closely related to ethylbenzene and alkylbenzene dioxygenases from *Rhodococcus* spp. (Figure 1). Based on these findings, we hypothesized that genes carried by this locus determine the degradation of unknown aromatic substrate(s) in a similar manner as with the model substances DF or BP, *i.e.* the aromatic ring is first activated by ARHD through dihydroxylation, and then is cleaved and further transformed (Seeger *et al.* 1995).

The architecture of the *iac* region resembles the eponymous locus in *Pseudomonas putida* 1290 enabling the catabolism of the plant auxin hormone and the signaling molecule indole-3-acetic acid (IAA; Scott *et al.* 2013). Expectably, the Pvy strain was able to utilize IAA as a sole carbon/energy source (Table 3).

The *mhp/vanAB/vdh* region consists of 22 coding sequences, including three putative regulatory protein- and four transporter protein-encoding genes. The products of genes here designated *vdh1* and *vanAB* exhibit high sequence similarity to vanillin dehydrogenase and two-component vanillate *O*-demethylase, respectively, from various pseudomonads. These enzymes transform the secondary metabolite vanillin to vanillic acid, which is further converted to protocatechuate. Vanillin dehydrogenases from various bacteria were reported to exhibit broad substrate specificity toward various aromatic aldehydes, such as isovanillin, veratraldehyde, anisaldehyde, protocatechualdehyde, *p*-hydroxybenzaldehyde, salicylaldehyde, and benzaldehyde (Masai *et al.* 2007; Mitsui *et al.* 2010; Graf *et al.* 2016; Nishimura *et al.* 2018). The genes *fadD* and *fadB* encode for a putative feruloyl-CoA synthase and vanillin synthase, respectively, enabling the catabolism of ferulic acid through its conversion to vanillin, which is further transformed by vanillin dehydrogenase and vanillate *O*-demethylase (Overhage *et al.* 1999). The predicted protein encoded by the *calA* gene exhibited high similarity with coniferyl-alcohol dehydrogenase from *Pseudomonas* sp. strain HR199 (Achterholt *et al.* 1998). Furthermore, the *mhp/vanAB/vdh* locus bears the *mhpABCDFE* gene cluster, with the *mhpA* gene encoding for a putative bifunctional 3-(3-hydroxy-phenyl)propionate/3-hydroxycinnamic acid monooxygenase that mediates the metabolism of 3-hydroxycinnamic acid. Moreover, an additional copy of a putative vanillin dehydrogenase gene, *vdh2*, was present within the *mhp/vanAB/vdh* locus, the predicted protein sequence of which exhibited a somewhat low relatedness to the vanillin dehydrogenases reported to date, including those from *P. putida* and *P. fluorescens* (66% and 57% of sequence similarity, respectively). Therefore, we predicted that the *mhp/vanAB/vdh* region determines the utilization/degradation of phenylpropanoids, presumably including lignin-derived compounds (Kamimura *et al.* 2017). The ability of the strain Pvy to utilize *trans*-cinnamic acid, vanillin, and vanillic acid was demonstrated; however, this was not true of caffeic acid or ferulic acid (Table 3). Predicted proteins encoded by three coding sequences corresponding to locus tags E4167_27585 and E4167_27590 show only low similarity to proteins with annotated functions available in the databases referenced. Nonetheless, domains specific for acyl-CoA dehydrogenase, acyl-CoA acetyltransferase, and putative phenol degradation protein were identified in the predicted amino acid sequences. The substrate specificity of corresponding putative enzymes and their potential involvement in aromatic compound degradation is unclear.

Two other ORFs encoding for putative Rieske-type ARHDs were found within the Pvy chromosome, here designated *ard2* and *ard3*. The amino acid sequences of the corresponding predicted α-subunits (locus tags E4167_29595 and E4167_29825) only exhibited a limited level of relatedness to characterized ARHDs available in databases. In the phylogeny reconstruction, the two predicted proteins formed a separate branch within a cluster belonging to ARHDs catalyzing the transformation of various aromatic substrates including NP, indene, and terephthalate (Figure 1). Surprisingly, at least some of the ORFs surrounding these putative ARHD-encoding genes indicated that these could be part of an aerobic non-specified aromatic compound catabolism pathway through CoA-thioesters, analogously as was reported for the *paa*-encoded pathway of phenylacetate degradation in both *Escherichia coli* K12 and *Pseudomonas* sp. strain Y2 (Teufel *et al.* 2010). Metabolic pathways that involve the putative ARHDs encoded by the *ard2* and *ard3* genes remain enigmatic, and discovering their exact function will require more research.

The analysis of the pND plasmid sequence revealed the presence of two ORF clusters here designated *ndo*/*nah* and *salA*/*dmp*, separated by a DNA span of 26.7 kbp. The predicted amino acid sequences encoded within the above-mentioned region showed a high similarity with proteins from *Pseudomonas* spp., putatively encoding a two-component NP 1,2-dioxygenase accompanied by a corresponding ferredoxin (genes *ndoBC* and *ndoA*, respectively), that initiate NP degradation. In the phylogeny reconstruction, the predicted protein sequence of the corresponding ARHD large subunit NdoB formed a cluster together with other NP, chloronitrobenzene, and nitrotoluene dioxygenases from the genera *Pseudomonas*, *Comamonas*, *Burkholderia*, and *Ralstonia* (Figure 1). In addition to the ARHD, other enzymes encoded within the *ndo/nah* cluster are homologous with those that enable subsequent reactions, which yield salicylic acid (genes *nahB*, *nahF*, *nahC*, *nahE*, *nahC*). The *ndo*/*nah* cluster also includes the *pahQ* gene, which encodes for a putative outer membrane porin involved in the metabolism of aromatic compounds.

The *salA*/*dmp* region is comprised of 12 genes, including those encoding for a putative transcriptional regulator (*nahR*), salicylate monooxygenase (*salA*), and the *dmp* cluster, the combination of which enables the catabolism of salicylate to pyruvate and acetylCoA through the *meta*-cleavage of the aromatic ring. A putative heme-binding protein of unknown function is encoded within this region (locus tag E4167_34205). Altogether, we hypothesized that the combination of enzymes encoded by the plasmid pND enables the utilization of NP through dihydroxylation with subsequent reactions leading to intermediates of the central metabolism. The ability of the Pvy strain to utilize NP and salicylate as sole carbon sources was confirmed experimentally within this study (Table 3).

### Induction of ARHDs by DF in resting cell assay

**Figure 2:**
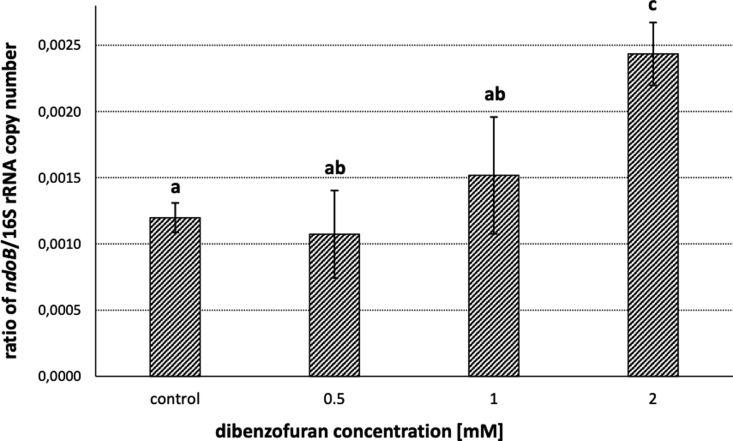
Induction of the transcription of NP-dioxygenase α-subunit (*ndoB*) gene by DF shown as the ratio of *ndoB* and 16S rRNA copy number. Error bars indicate standard error of the mean; bars sharing the same letter (a, b, c) are not significantly different (*P*-value <0.05); control: no DF added.

The DF degradation pathway documented in different bacterial strains produces salicylate (Bunz and Cook 1993; Armengaud and Timmis 1997), which is also an intermediate product in the degradation pathway of NP in *Pseudomonas* strains (Eaton and Chapman 1992). At the same time, the dioxygenation of DF has been reported to be catalyzed by DF 4,4a-dioxygenase (Bunz and Cook 1993; Kasuga *et al.* 2001), but due to their broad substrate specificity, other ARHDs have been found to catalyze its dioxygenation, such as BP dioxygenase (Seeger *et al.* 2001; Mohammadi and Sylvestre 2005; L’Abbée *et al.* 2005) or NP 1,2-dioxygenase (Resnick *et al.* 1996), which is encoded in the plasmid pND of Pvy. With all of this in mind, and due to the fact that the bacterium Pvy was isolated as a DF-degrading bacterium, we further investigated whether the predicted plasmid-borne NP 1,2-dioxygenase large subunit is involved in DF degradation. For this purpose, the induction of the corresponding large subunit-encoding gene *ndoB* in the presence of DF was determined. The results of the qPCR with cDNA obtained from DF-exposed Pvy cultures revealed significant differences in the levels of *ndoB* transcripts (Figure 2). There was no significant difference between the control (no DF added) and 0.5 mM DF, and the difference between the control and 1 mM was marginally insignificant (*P* = 0.06). Importantly, there was a significant difference between the control and 2 mM DF (*P *<* *0.001). Hence, we assume that the observed DF degradation by the Pvy strain is mediated by the putative NP-dioxygenase encoded by the plasmid-borne gene cluster *ndoABC*.

In addition to *ndoB*, the induction of the dioxygenases of unknown function *ard1*, *ard2*, and *ard3* was assessed to determine their possible involvement in DF degradation. Since the deduced aa sequences of *ard2* and *ard3* exhibited a significant level of similarity (82%), and in the phylogeny reconstruction formed a discrete cluster (Figure 2), only *ard2* as a representative of both was followed in this study. Nevertheless, unlike with *ndoB*, no significant changes in the transcript levels of either *ard1* or *ard2* were observed, with *c*_p_ values not differing from the non-template control.

The transcription of NP degradation genes has been reported to be induced by salicylate, NP, or both of them (Schell 1983; Jones *et al.* 2003; Tomás-Gallardo *et al.* 2014). DF has been reported to induce ARHDs such as NarA in *Rhodococcus opacus* (Kimura *et al.* 2006), DfdA in *Terrabacter* sp. strain YK3 (Iida *et al.* 2002), or as we report here, Pvy-borne NdoB. Our results also demonstrated that the induction of biodegradative genes was concentration dependent. Higher concentrations of DF resulted in higher induction (Figure 2), while concentrations of 0.5 mM DF did not induce the gene significantly compared to the control (no DF added). These results are in agreement with the conclusions of other studies; for example Pham *et al.* (2015) found that the amount of 4-chlorobenzoate (an end metabolite of the *Rhodococcus erythropolis* U23A BP catabolic pathway) produced by cells co-metabolically grown on BP plus sodium acetate decreased while decreasing BP concentration. A similar result was found by Toussaint *et al.* (2012), where cells grown on sodium acetate and BP decreased their catabolic activity toward 4-chlorobiphenyl as the concentration of BP decreased.

## Conclusions

The analysis of the whole-genome sequence and experiments made with the Pvy strain support the conclusion that this strain is a versatile degrader of aromatic compounds. Introducing the strain or its DNA into the genetic pool of autochthonous microbial populations could potentially contribute to the microbe-assisted bioremediation of sites contaminated with various aromatic hydrocarbons. In addition, several genetic elements, including ARHD-like genes, were identified with low similarity to any known genes/enzymes, requiring their further investigation in order to fully clarify their roles. Furthermore, given the fact that the strain is capable of degrading both pollutants and plant-derived aromatic compounds, it could be employed in the plant-assisted bioremediation (Pham *et al.* 2015; Musilová *et al.* 2016; Vergani *et al.* 2019) of sites with high concentrations of pollutants that the strain is able to tolerate.
